# EUS-guided fine needle aspiration provides an open view for duodenal obstruction caused by urothelial carcinoma: a case report

**DOI:** 10.1186/s12876-022-02452-1

**Published:** 2022-08-08

**Authors:** Xiaoli Chen, Xin Chen, Xiaoli Yu, Xingkang He

**Affiliations:** 1 Division of Gastroenterology, School of Medicine, Sir Run Run Shaw Hospital, Zhejiang University, Hangzhou, China; 2 Division of Pathology, School of Medicine, Sir Run Run Shaw Hospital, Zhejiang University, Hangzhou, China

**Keywords:** EUS-FNA, Duodenal wall thickening, Urothelial carcinoma, Metastasis

## Abstract

**Background:**

Endoscopic ultrasound-guided fine-needle aspiration (EUS-FNA) is a good alternative and diagnostic tool for gastrointestinal wall thickening with prior negative endoscopic biopsies.

**Case presentation:**

Here we reported a case of a 60-years-old woman admitted with atrophic right kidney and hydronephrosis and intermittent postprandial bloating. Esophagogastroduodenoscopy and small bowel endoscopy revealed wall thickening and stenosis at the junction of the descending and inferior duodenum. Biopsies from endoscopy showed no specific findings. EUS-FNA of the thickened duodenal wall was performed and histopathological examinations revealed poorly differentiated carcinoma. Immunohistochemically staining was positive for pan-cytokeratin, CK7, CK20, and weakly positive for GATA-3 and P63. These results were highly suggestive of metastatic urothelial cancer.

**Conclusions:**

EUS-FNA played an important role in the diagnosis of unexplained gastrointestinal wall thickening and rare metastases to the gastrointestinal wall.

## Background

Gastrointestinal wall thickening could be mostly observed in the stomach, esophagus, and rectum [1]. A variety of pathologies, including both benign and malignant causes could lead to the thickening of the gastrointestinal tract [2, 3]. Broadly speaking, benign causes include inflammatory, autoimmune, infectious, infiltrative diseases and malignant causes include cancer, lymphoma, and metastasis [3, 4]. Duodenal wall thickening is a non-specific finding in abdomen imaging. The differential diagnosis of duodenal wall thickening is quite broad and difficult. The accurate diagnosis was mostly based on pathological examination and was essential for treatment options. However, conventional biopsies from endoscopy were always falsely negative, especially for submucosal infiltrating cancer. Therefore, identifying the cause of duodenal wall thickening remains a challenge for clinicians. Recently, with development of endoscopic ultrasound-guided fine needle aspiration (EUS-FNA), it emerged as the important tool to obtain samples to make a definitive diagnosis.

Here we reported a case of a 60-year-old woman with an atrophic right kidney and hydronephrosis. EGD revealed duodenal wall thickening and stenosis. Biopsies from EGD showed no specific findings. Finally, EUS-FNA was adopted and histological results revealed tumor nests in the duodenal wall. The primary diagnosis of urothelial carcinoma was determined based on an immunohistochemical study.

### Case presentation

**Fig. 1:**
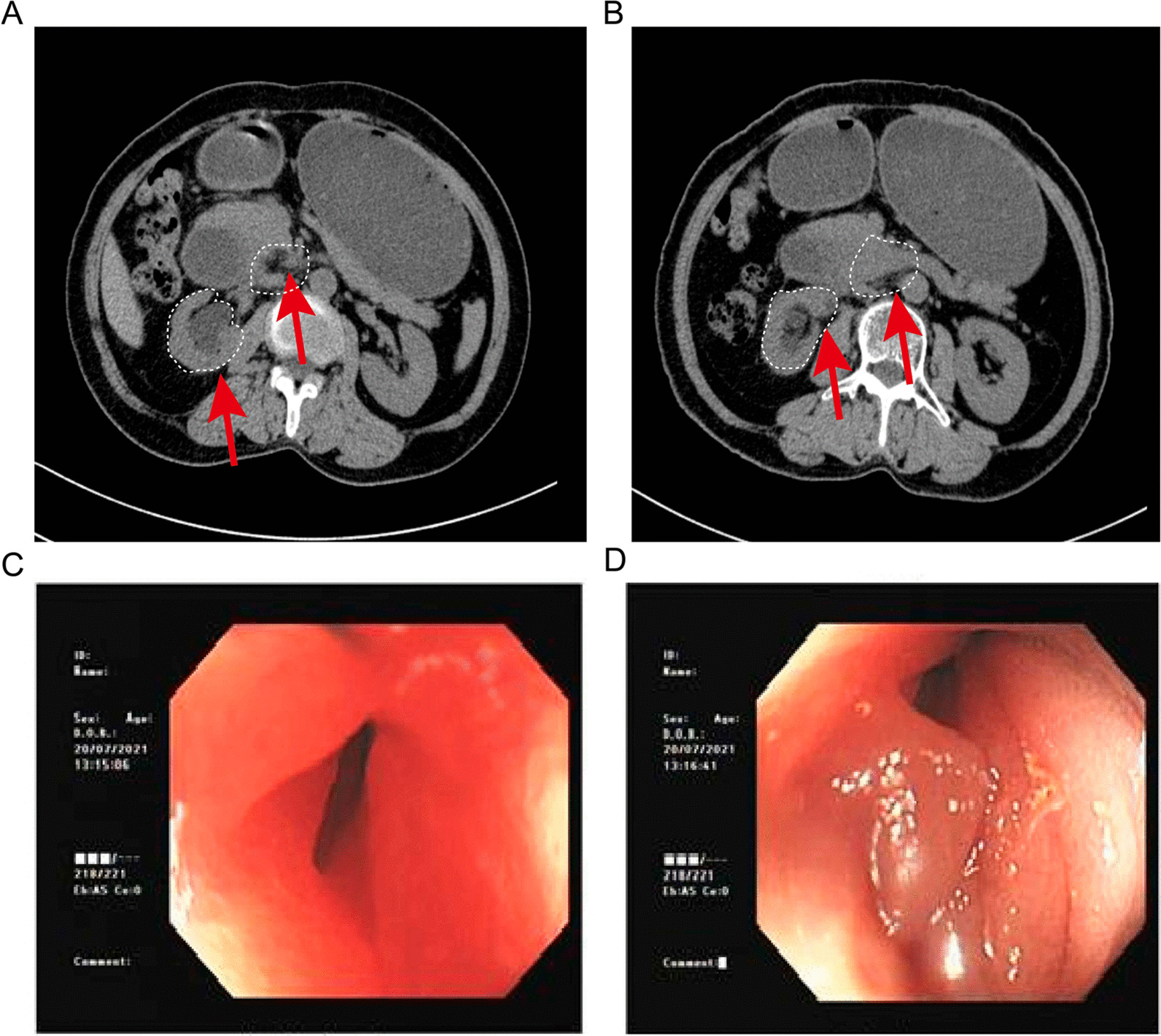
**A** and **B**, Computed tomography showed left hydronephrosis and thickening of the descending duodenum. **C** and **D**, Esophagogastroduodenoscopy (EGD) and small bowel endoscope revealed wall thickening and stenosis of the duodenum

**Fig. 2:**
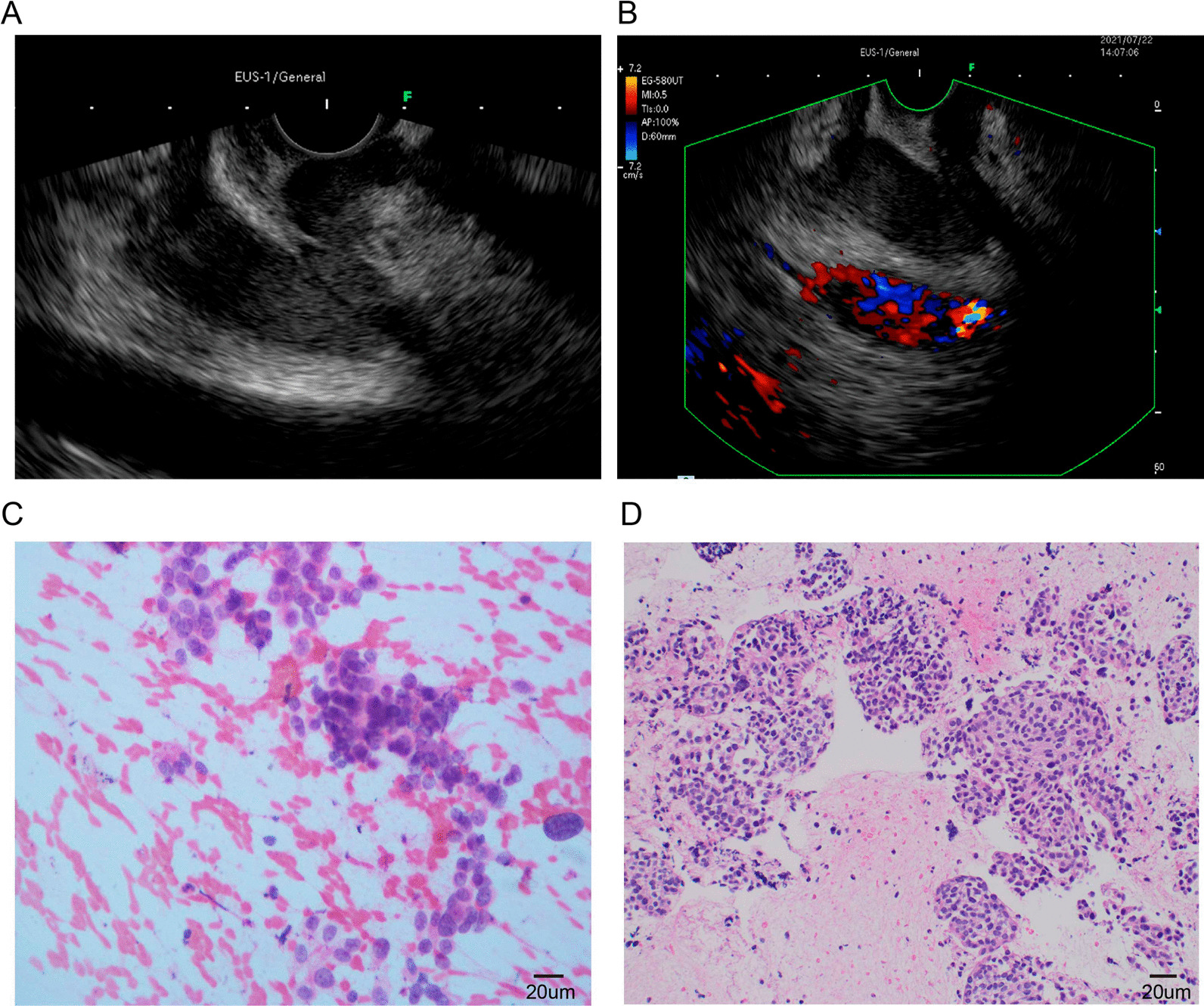
**A** and **B**, Endoscopic ultrasound (EUS), and Doppler EUS revealed duodenal thickening. **C**, Cytology of endoscopic ultrasound-guided fine-needle aspiration (EUS-FNA) specimens (Nikon DS-U3, 40X). **D**, Hematoxylin and eosin staining of EUS-FNA specimens (Nikon DS-U3, 20X)

**Fig. 3:**
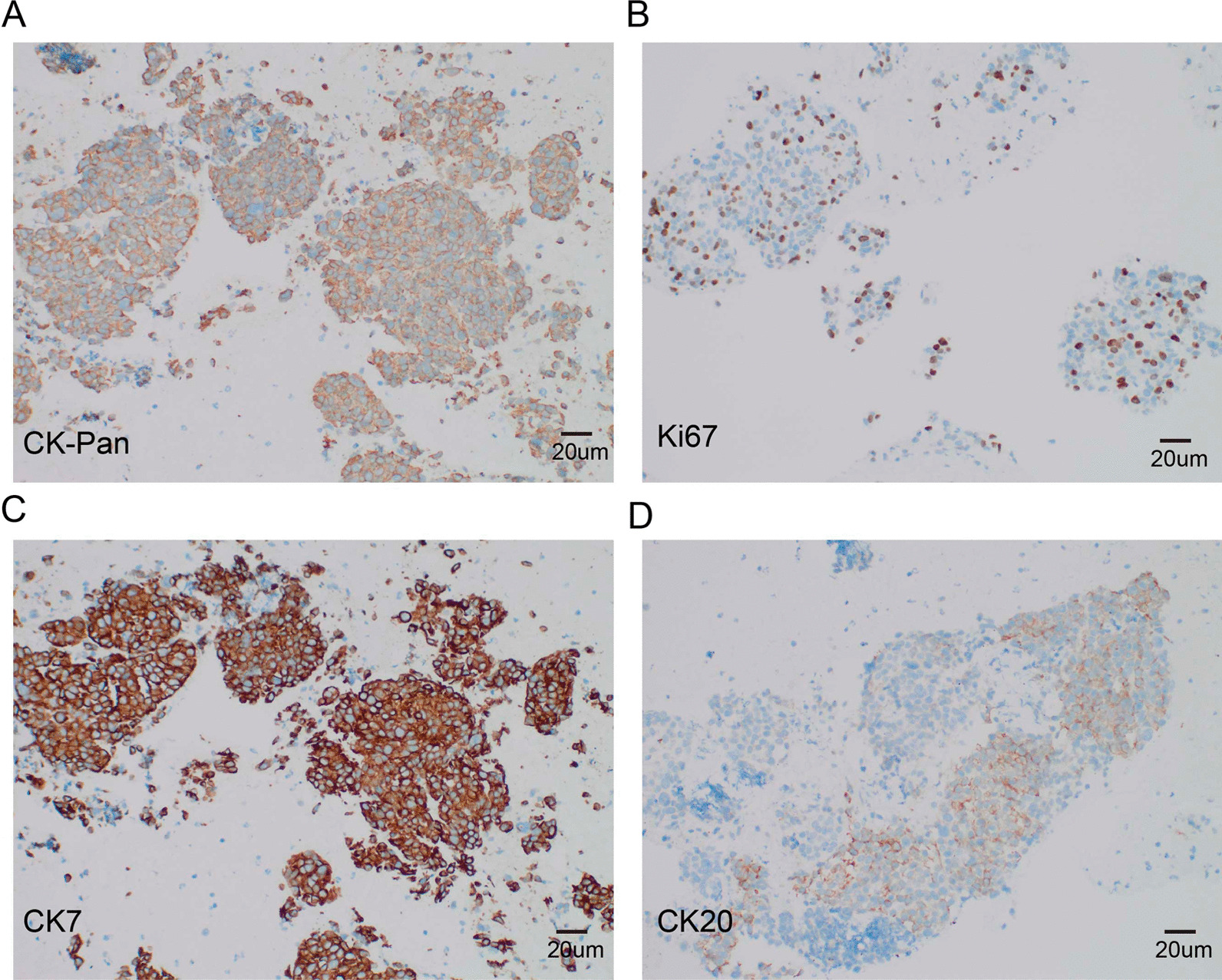
The immunostaining findings of EUS-FNA specimens are as follows: **A**, Cytokeratin (CK)-Pan staining (Nikon DS-U3, 20X); **B**, Ki-67 staining (Nikon DS-U3, 20X); **C**, Cytokeratin7 (CK7) staining (Nikon DS-U3, 20X); **D**, Cytokeratin20 (CK20) staining (Nikon DS-U3, 20X)

**Fig. 4:**
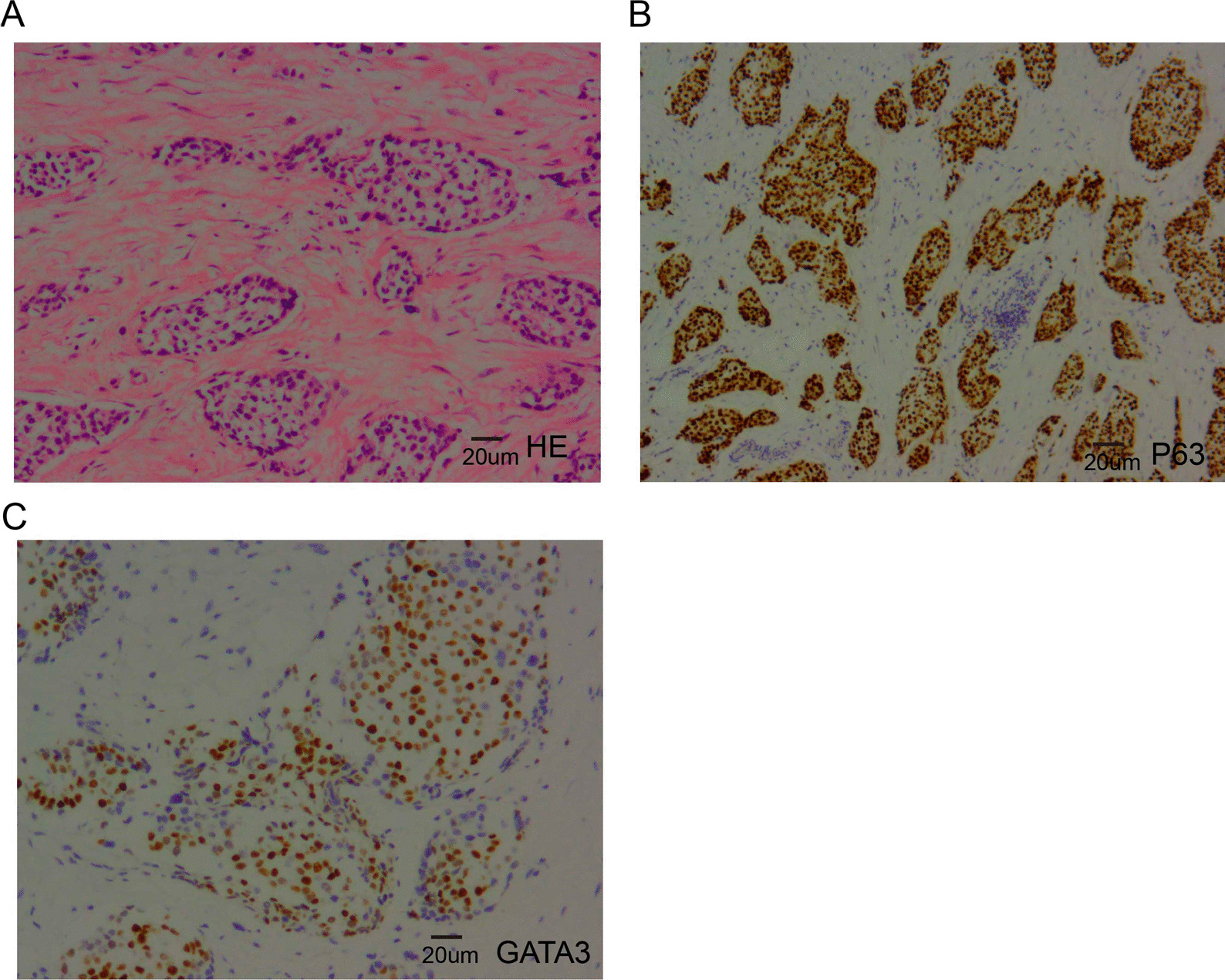
The immunostaining findings of surgical specimens are as follows: **A**, Hematoxylin and eosin staining (Nikon DS-U3, 20X); **B**, Tumor protein 63 (P63) staining (Nikon DS-U3, 20X); **C**, GATA binding protein 3 (GATA3) staining (Nikon DS-U3, 40X)

A 60-year-old woman with a past medical history of hypertension was admitted to the hospital with complaints of atrophic right kidney with hydronephrosis and intermittent postprandial bloating. A physical examination revealed left lower quadrant abdominal tenderness and no costovertebral angle tenderness. A laboratory examination revealed increased serum levels of creatinine. No other abnormal findings were observed in urine analysis and autoimmune disease tests. Abdominal computed tomography (CT) showed wall thickening of the descending part of the duodenum and left hydronephrosis with atrophic renal parenchyma (Fig. 1A, B). Since the patient was allergic to procaine and iodine, contrast-enhanced CT could not be performed. Consequently, EGD and small bowel endoscopy were performed, and these tests revealed circumferential stenosis at the junction of the descending and inferior duodenum (Fig. 1C, D). Biopsies from EGD and small bowel endoscope were obtained, and histopathological examination only revealed duodenitis. Based on these findings, the underlying cause of the duodenal wall thickening remained unclear since no specific findings. To identify the underlying reason, EUS-FNA of the thickened duodenal wall was successfully performed with a 22 G needle (Cook Medical, USA). EUS of the duodenal lesion showed a thickened duodenal wall (thickness: 15 mm, Fig. 2A, B). On-site evaluation for a poorly carcinoma is made because of increased cellularity and markedly atypical clusters. Further immunohistochemical analysis revealed that the cancer cells were positive for CK-Pan, cytokeratin 7 (CK7), cytokeratin 20 (CK20), and partly positive Ki-67 (Fig. 3). Based on immunohistochemical stating, we suspected that poorly differentiated carcinoma was spread from the urinary system. Due to obstruction of the urinary tract and the duodenum, the patient received a ureteric stent and gastrointestinal bypass surgery. Biopsy specimens were also obtained from the procedure. The final pathological diagnosis of urothelial carcinoma was made based on P63-positive and GATA3-positive (Fig. 4B, C), which was consistent with the initial diagnosis of EUS-FNA.

## Discussion and conclusion

Urothelial carcinoma (UCC) is the most common type of bladder cancer and common symptoms of UCC are hematuria and back pain [5]. Urine cytology and cystoscopy are the gold standards in the diagnosis of UCC [6]. Approximately 20% of patients with invasive UCC will develop metastatic diseases [7]. Lymph node metastasis and involvement in UCC were quite common and UCC usually metastasizes to distant organs, such as the lung, liver, stomach, skin, and eyes [8–12]. Several case reports have described that UCC could metastasize to the duodenum [13–17]. Duodenal malignant was extremely rare and duodenal adenocarcinoma was a primary tumor for malignant disease. Duodenal metastasis could result from other organs, including the breast, lung, kidney, prostate, liver, colon, and uterus [18–20]. The thickness of the duodenal wall in the current study was quite large and biopsies from conventional endoscopy were negative. Thus the current diagnosis of duodenal wall thickening or stenosis remained a challenge for clinicians when CT did not identify a primary site or endoscopic biopsy revealed no specific findings. The present case highlighted that EUS-FNA might be an indicative, and minimally invasive way to obtain adequate samples for diagnosis of duodenal thickening of unknown cause. EUS-FNA was initially adopted by Vilmann et al. for diagnosis of pancreatic cancer [21] and subsequently became an important diagnostic tool for gastrointestinal lesions. EUS-FNA was considered the gold standard for staging and diagnosis of gastrointestinal malignancies since its high sensitivity and specificity [22]. Furthermore, EUS-FNA could puncture extra-luminal lesions from the gastrointestinal tract to provide additional histological evidence. European society of gastrointestinal endoscopy also suggested performance of EUS-guided sampling after failure of standard biopsy techniques [23]. Actually, the performance of EUS-FNA in diagnosis of unexplained thickening of the esophagogastric and stomach wall had been well established. For the esophagogastric wall, nine of ten patients were diagnosed correctly without complications using EUS-FNA [24]. In cases of stomach disease, the diagnostic accuracy of EUS-FNA for linitis plastica was 87.5% without severe hemorrhage or perforation [25]. There were no severe complications associated with the procedure in this setting, suggesting the safety of EUS-FNA. EUS-FNA has been well demonstrated to be a safe technique with relatively low morbidity and mortality rates[26]. The majority of complications associated with EUS-FNA included perforation, hemorrhage, acute pancreatitis, and infection [27]. According to a previous systematic review, the complication rate and the mortality rate were approximately 1–0.98% [28].

However, the application of EUS-FNA for duodenal lesions remained rare. One reason might be technically challenging for EUS-FNA. Due to special training and a long learning curve, EUS-FNA was considered a difficult technique to master [29]. Our case showed the usefulness of EUS-FNA in the diagnosis of unknown wall thickening of the duodenum. Previously, five cases reported the diagnosis of UTUC with duodenum involvement [14–17, 30]. Only two of them were diagnosed by EUS-FNA [14, 15], and three cases were made by surgery or autopsy [16, 17, 30]. According to a previous study, EUS-FNA was rarely used to diagnose lesions of duodenal mass [31]. In the current case, samples from EUS-FNA provided important cytological evidence for further treatments. However, tissues from EUS-FNA were limited and sometimes were unable to provide enough material for correct diagnosis. To overcome this limitation, EUS-fine needle biopsy (FNB) was developed. Recently, one study reported that EUS-fine needle biopsy (FNB) technique had excellent diagnostic performance and safety in the study of unexplained diffuse gastrointestinal wall thickening [1]. We, therefore, suggested EUS-FNA/FNB should be performed in cases with prior negative endoscopic biopsies for the diagnosis of unexplained thickening of the duodenum.

In conclusion, we reported a case of EUS-FNA that helped to diagnose UCC with duodenal metastasis. For unexplained thickening of the duodenal wall, the accurate diagnosis is necessary for further suitable treatments. In this sense, EUS-FNA can be an effective method for providing clues or achieving a diagnosis.

## Data Availability

All data used in current study are included in the published article.

## Abbreviations

EUS-FNA: Endoscopic ultrasound-guided fine needle aspiration
EGD: Esophagogastroduodenoscopy
CT: Computed tomography
UCC: Urothelial carcinoma
EUS-FNB: Endoscopic ultrasound-guided fine needle biopsy
